# Intractable Fasting Hypoglycemia as a Manifestation of Hepatocellular Carcinoma

**DOI:** 10.1155/2017/7465025

**Published:** 2017-07-12

**Authors:** Justin J. Forde, Ofor Ewelukwa, Tony Brar, Roniel Cabrera

**Affiliations:** ^1^Department of Medicine, University of Florida, 1600 SW Archer Road, Gainesville, FL 32610, USA; ^2^Division of Gastroenterology, Hepatology and Nutrition, University of Florida, 1600 SW Archer Road, Gainesville, FL 32610, USA

## Abstract

Non-islet cell tumor hypoglycemia (NICTH) is a rare and serious paraneoplastic complication of both malignant and benign tumors to consider when evaluating fasting hypoglycemia, especially in the setting of liver diseases. We present a case of NICTH in a 54-year-old male with hepatocellular carcinoma (HCC) who presented with symptomatic intractable hypoglycemia (IH) after bowel preparation and fasting for screening upper endoscopy and colonoscopy.

## 1. Introduction

Hypoglycemia due to non-islet cell tumor hypoglycemia (NICTH) is an established paraneoplastic complication of hepatocellular carcinoma (HCC) that occurs in 4–27% of patients [1–3]. The actual prevalence and incidence are not known, likely due to a large number of cases that go unrecognized. Rarely, NICTH has been identified as the initial symptom of HCC in otherwise asymptomatic individuals [1, 4]. There are two types based on main etiologies for NICTH, types A and B. Type B is more common and is caused by overproduction of incompletely processed insulin-like growth factor 2 (IGF-2) by the tumor cells. Less common is type A, characterized by increased glucose demand from rapidly growing tumor, late in the disease process. Both types result in intractable fasting hypoglycemia and should be considered in patients with refractory hypoglycemia [1, 5–8]. NICTH occurs more commonly in patients with mesenchymal tumors, fibromas, carcinoid, myelomas, lymphomas, hepatocellular, and colorectal carcinomas [5, 9, 10].

## 2. Case

A 54-year-old male with long-standing hepatitis C virus related cirrhosis, and advanced HCC with portal vein (PV) invasion, diagnosed two months prior to presentation by CT scan and core needle biopsy, was evaluated in the emergency department for dizziness, incoherent speech, agitation, and diaphoresis after completing bowel preparation and fasting for colonoscopy. The patient was initially found to have a blood glucose level of 22 mg/dL, which corrected to normal limits after administration of dextrose. The patient was discharged from the emergency department in stable condition; however on presentation for endoscopy later that day the patient complained of visual changes and was noted to have slurred speech. Blood glucose at that time was 20 mg/dl and the patient was admitted for further evaluation and management of severe hypoglycemia. The patient was treated with continuous dextrose 10% infusion with titration and high carbohydrate diet; however symptomatic fasting hypoglycemia persisted requiring frequent IV dextrose 50% pushes. Insulin level was 0.37 mcIU/mL and the C-peptide level was 0 ng/mL, suggesting a non-islet cell etiology. Surreptitious insulin use was excluded with regular monitoring. His home medications of Tramadol and Lexapro were discontinued on admission due to their potential for hypoglycemia. Adrenal insufficiency was excluded with cortisol stimulation test and a pancreatic protocol CT of the abdomen showed no pancreatic mass. There were no signs or symptoms of sepsis to suggest infectious etiology of this patient's hypoglycemia and infectious workup, including urinalysis, stool culture, and chest X-ray, was negative. Diagnostic paracentesis was not possible due to negligible ascites accumulation. The patient was subsequently started on daily oral prednisolone, with some improvement in the severity of hypoglycemia; however due to frequent episodes of hypoglycemia, continuous dextrose could not be discontinued. A trial of octreotide at 25 mcg/hr was attempted; however the patient continued to have hypoglycemia and this was discontinued after 7 hours due to concerns for potential worsening hypoglycemia. IGF-2 levels eventually resulted as 220 ng/mL, making further trials of octreotide therapy unnecessary.

The patient was not a candidate for surgical or palliative cytoreductive therapies due to the large tumor size with PV infiltration, poor performance status, and active symptoms. Blood glucose concentrations were eventually stabilized with continuous dextrose 30% infusion, frequent high glucose meals, and prednisolone 30 mg twice a day; however, he later began to deteriorate and developed massive upper gastrointestinal bleed, likely from esophageal varices. The patient eventually opted for supportive comfort care and died in the hospital nearly one month after his initial presentation.

## 3. Discussion

Two main types of NICTH have been described, differentiated by their pathophysiology and timing. Type A is characterized by high tumor utilization of glucose in poorly nourished patients with depleted glycogen stores and defective gluconeogenesis. This type of NICTH is often seen during the late stages of HCC when tumor burden is large and hepatic destruction is extensive [9, 10]. In these patients, hepatic regulation of blood glucose levels via gluconeogenesis and glycogenolysis is significantly impaired. They have low insulin and C-peptide levels with high levels of glucagon and other counterregulatory hormones [5].

In type B NICTH, there is increased tumor secretion of incompletely processed IGF-2 (pro-IGF-2), which is poorly metabolized due to defective hepatocytes in cirrhosis. This defective pro-IGF-2 is smaller, crosses the capillary membranes easier, and stimulates more insulin receptors throughout the body than normal IGF-2. This occurs early in liver disease and is characterized by overwhelming tissue glucose uptake and severe, persistent hypoglycemia [8, 11–14]. Our patient was negative for insulin autoantibodies, had low levels of IGF-2, insulin, and C-peptide, and had high glucagon levels, making his condition more likely due to type A NICTH.

Acute management of the condition is usually similar to that of hypoglycemia during the treatment of diabetes with carbohydrate and glucagon by subcutaneous or intramuscular injections in patients who are unable to tolerate oral carbohydrate [5, 15]. Our patient failed to maintain adequate glucose levels despite continuous infusion of dextrose at varying rates.

Treatment aimed at the underlying malignancy such as cytoreductive surgery is the mainstay especially in type B NICTH where complete removal of the tumor can often lead to resolution of hypoglycemia [12]. It is effective at increasing survival but has a 7.1% in-hospital procedure mortality rate [16]. Despite moderate benefit, this strategy often proves difficult or impossible as large tumors are often unresectable and patients are often too ill and therefore poor surgical candidates. Surgery was not possible in our patient due to large tumor size and infiltration of the PV. Trial of percutaneous ethanol injection of the tumor was successful in resolving hypoglycemia in one case [17]. Chemotherapy, radiation, and selective embolization are other therapeutic options to be considered [18]. Patients with HCC have a survival benefit with transarterial chemoembolisation (TACE) when appropriately selected, which has also been used in those with HCC-induced hypoglycemia with favorable outcomes [19].

In the event that the underlying malignancy cannot be treated, medical management is used to prevent hypoglycemia. Steroids, glucagon infusions, octreotide, and growth hormone are commonly utilized, with varying results, particularly in patients with poor response to carbohydrates [1, 9, 15, 20]. Glucocorticoids are considered an effective means of long-term management of hypoglycemia by suppressing IGF-2, stimulating gluconeogenesis, and modulating the growth hormone-IGF axis [15, 21]. This may be much less effective in the setting of poor hepatic reserve from cirrhosis, or in decreased hepatic function from high tumor burden.

In conclusion, NICTH should be considered in the evaluation of intractable fasting hypoglycemia, particularly in the setting of chronic liver disease and in patients with risk factors for HCC. Patients may be unaware of hypoglycemia, particularly after recurrent hypoglycemic episodes when awareness begins to diminish. NICTH has a poor prognosis and high rates of treatment failure, despite aggressive treatment modalities. The mainstay of therapy is decreasing tumor burden through resection or embolization, but this may not be possible due to tumor size or infiltration of nearby structures. Glucocorticoids, frequent high carbohydrate meals, and intravenous carbohydrate infusions may help, but these methods have mixed results. Awareness of the existence of this clinical entity may help in early diagnosis and possible prevention of NICTH in at-risk populations.
